# Bringing nature into decision-making

**DOI:** 10.1098/rstb.2022.0313

**Published:** 2024-06-10

**Authors:** Yadvinder Malhi, Gretchen C. Daily

**Affiliations:** ^1^ Environmental Change Institute, School of Geography and the Environment, University of Oxford, Oxford OX1 3QY, UK; ^2^ Leverhulme Centre for Nature Recovery, University of Oxford, Oxford OX1 3QY, UK; ^3^ Natural Capital Project, Stanford University, Stanford, CA 94305, USA; ^4^ Department of Biological Sciences and Woods Institute for the Environment, Stanford University, Stanford, CA 94305, USA

Contemporary economic development has led to dramatic decline of the natural world according to a wide range of metrics. There is also powerful evidence and growing recognition that this decline matters—not only because of the intrinsic value of Earth's biodiversity, but also because the degradation of the web of life threatens human well-being today, social and economic progress, and even the future of our civilization. A widespread diagnosis of this predicament is that the value and importance of the natural world is not sufficiently accounted for in economic or other decision-making processes, and that bringing nature into decision-making provides the potential for a systemic solution to this challenge. Recent developments and statements have raised awareness of the biodiversity crisis, resulting in high-level calls to ‘bend the curve’ of biodiversity loss within a decade, and to create nature-positive economies and businesses. New policy impetus has come from the adoption of the 2022 Kunming-Montreal Biodiversity Framework, and well as a flurry of new nature-focused legislation at supernational, national and city levels.

How exactly bringing nature into decision-making can be achieved across sectors and at scale remains a major challenge. However, there has been substantial progress in developing successful demonstrations of integrating nature into decision-making in a variety of sectors and regions, and an increasing number of approaches to the challenge of scale.

In this thematic issue, we examine some of the challenges and most promising solutions for bringing nature into decision-making at scale, highlighting successful demonstrations and cases in a variety of sectors.

Vatn *et al.* [1] kick off the collection with an exploration of what values we assign to nature, and how these values play an important role in shaping decision-making. There are multiple values of nature but a narrow few have tended to dominate the incorporation of nature into decision-making, e.g. instrumental financial value in payments for ecosystem services, or intrinsic values in the establishment of protected areas. Vatn *et al.* present a framework for analysing which values are prioritized and which are left out. They analyse key areas of environmental policy, including the development of the present model for nature protection since the mid-twentieth century, the role that valuation plays in bringing nature's values into decisions, and the values embedded in two key environmental policy instruments: protected areas for nature and payments for ecosystem services. Their analysis highlights the inherent tension in how environmental policies have been established as minor additions to decision-making structures that prioritize economic expansion, which tends to further undermine a wide range of nature's values.

A suite of papers then examines the potential of ecosystem services valuation and natural capital accounting as pragmatic means for bringing nature into decision-making. They consider both monetary approaches, for services such as carbon storage, provision of opportunity for recreation and flood damage mitigation, as well as non-monetary approaches to valuing nature, especially non-material and relational dimensions of biodiversity.

Umaña Quesada [2] gives an example of how governments have brought nature into decision-making, providing an overview of how Costa Rica created a pioneering and system for giving value to standing forests, drawing on his first-hand experience as Minister for Environment and Energy in the late 1980s, charged with designing and implementing this policy. The policy was centred on fiscal incentives for reforestation, financed by an international debt-for-nature swap mechanism.

Transnational corporations are major agents whose decision-making practices can have positive or negative outcomes on nature. Bebbington *et al.* [3] explore how such corporations attempt to take nature into account in their decision-making. They identify three critical success factors that need to be satisfied: a robust way to link local nature outcomes with company activities, translation of company strategic intentions with respect to nature into operational routines that inform actions on the ground, and financial actors creating the right incentives in support of company decision-making.

Major recent advances in traceability and global information systems provide new opportunities for addressing these critical factors. Natural capital accounting provides one such data-rich approach that can be used by government and businesses to assess the state of nature, their impacts on nature and their dependencies on nature. Ninety-two countries are now committed to developing national natural capital accounts to increase integration of the values of nature into decision-making. Notably, this list now includes the USA, a significant development given the influence of its economy. The potential of such national natural capital accounting to support businesses in their requirements and efforts to report on their nature impacts is highlighted and explored by Ingram *et al*. [4]. Next, Day *et al.* [5] examine how natural capital approaches can practically inform design of nature recovery policies, with a case example of policies incentivizing the creation of new natural habitat in England.

Vasseur & Andrade [6] present a case study from the Colombian Andes of how assessment tools and standards can be applied to support decision-making in identifying at-risk ecosystems and supporting their social-ecological resilience through application of ecosystem-based adaptation. They describe and apply two tools that were developed by the International Union for Conservation of Nature: the Red List for Ecosystems and the Nature-Based Solutions Global Standard, and show how these global tools can support locally nuanced decision-making.

With more than half of the world's population living in urban environments, and that proportion increasing as the twenty-first century unfolds, cities are a key nexus for bringing nature into decision-making, enhancing biodiversity, supporting climate change adaptation and maintaining human connections to the natural world. Chan [7] provides examples of 11 cities and how they have enhanced urban nature, and explores what lessons can be learned for replicating and scaling up models of success.

Hazell & Clarke [8] also explore the topic of enhancing nature connection, in this case in the context of children's exposure to nature in the school environment. They review the literature that shows enhanced benefits for children's physical and mental health, focus at school and nurturing of pro-nature attitudes. They then examine the UK Government's National Education Nature Park, planned across all schools and kindergartens as a strategy for increasing nature connection in educational curriculum design and decision-making.

Finally, Anderies & Folke [9] explore deeper questions of the need for meaning and purpose in our relationships with the biosphere, beyond practical tools for nature valuation and accounting. They ask what collective shared stories—imagined orders—we can tell ourselves that enable us to revitalize meaningful and nurturing relationships with nature and the global biosphere in the Anthropocene. They argue that incremental adjustment of existing institutions and organizations is unlikely to be sufficient to address the current global-scale challenge of our broken relationship with nature. They offer no straightforward solutions but try to sketch out some of the elements of such a collective story. These elements include recognition of global intertwining between humanity and the biosphere, navigating human actions and societies within a global safe operating space, active stewardship encouraged by appreciation of and experiencing and the unique context of life on Earth, and recognition of contemporary and intergenerational justice.

As a collection, these papers highlight the urgency of bringing nature into decision-making, and also demonstrate the real progress in application of data rich tools that facilitate such decision-making, while at the same time reminding us that there are deeper issues around our relationship with the straining biosphere that need examination and remedying.

Most of the papers draw on a UK-US Forum that was held in June 2022 at the Royal Society, that invited a unique breadth of participants from academia, business, government and other sectors to examine these issues.

## Data Availability

This article has no additional data.
